# Biomaterial Studies on AISI 316L Stainless Steel after Magnetoelectropolishing

**DOI:** 10.3390/ma2010129

**Published:** 2009-03-11

**Authors:** Tadeusz Hryniewicz, Krzysztof Rokosz, Massimiliano Filippi

**Affiliations:** 1 Koszalin University of Technology, Division of Surface Electrochemistry, Raclawicka 15-17, PL 75-620 Koszalin, Poland; E-Mail: Krzysztof.Rokosz@tu.koszalin.pl; 2 Plasma and Advanced Materials, Fondazione Bruno Kessler – irst, I-38050 Povo (Trento), Italy

**Keywords:** 316L stainless steel, magnetoelectropolishing, polarization curves, SEM, Auger spectroscopy, XPS

## Abstract

The polarisation characteristics of the electropolishing process in a magnetic field (MEP – magnetoelectropolishing), in comparison with those obtained under standard/conventional process (EP) conditions, have been obtained. The occurrence of an EP plateau has been observed in view of the optimization of MEP process. Up-to-date stainless steel surface studies always indicated some amount of free-metal atoms apart from the detected oxides and hydroxides. Such a morphology of the surface film usually affects the thermodynamic stability and corrosion resistance of surface oxide layer and is one of the most important features of stainless steels. With this new MEP process we can improve metal surface properties by making the stainless steel more resistant to halides encountered in a variety of environments. Furthermore, in this paper the stainless steel surface film study results have been presented. The results of the corrosion research carried out by the authors on the behaviour of the most commonly used material − medical grade AISI 316L stainless steel both in Ringer’s body fluid and in aqueous 3% NaCl solution have been investigated and presented earlier elsewhere, though some of these results, concerning the EIS Nyquist plots and polarization curves are also revealed herein. In this paper an attempt to explain this peculiar performance of 316L stainless steel has been undertaken. The SEM studies, Auger electron spectroscopy (AES) and X-ray photoelectron spectroscopy (XPS) were performed on 316L samples after three treatments: MP – abrasive polishing (800 grit size), EP – conventional electrolytic polishing, and MEP – magnetoelectropolishing. It has been found that the proposed magnetoelectropolishing (MEP) process considerably modifies the morphology and the composition of the surface film, thus leading to improved corrosion resistance of the studied 316L SS.

## 1. Introduction 

For years, several positive features concerning electropolished metal surface have been ascribed to the so called *plateau* of the polarisation curve. The use of an externally applied magnetic field to the electropolishing process (EP) provides the treated metal surface with some new properties and better characteristics, including microroughness, hydrophilicity, contact angle, corrosion resistance, and oxide film morphology [1,2,3,4,5,6]. A higher level of metal finishing is attractive in multiple applications, such as, e.g., for medical stents and implant devices [1,7,8,9,10,11,12,13,14,15,16]. The addition of an external magnetic field to the process of electropolishing also significantly minimizes microtopography by lowering microroughness and minimizing the actual surface area on the micro- and nano scales [1,2,3,5,7,8,9,10].

Type UNS S31603 316L stainless steel is a low-carbon version of the AISI 316 stainless steel used extensively for many purposes due to its superior corrosion resistance, smoothness, biocompatibility and cleanability after electropolishing treatment [17,18,19,20]. The remarkable improvement in corrosion resistance of electropolished surfaces of austenitic stainless steels is caused by several interconnected events occurring during the electropolishing process, discussed extensively elsewhere [3,9,10,11,12,13,14,15,20,21]. Thus, the obtained stainless steel exhibits excellent corrosion resistance in a wide range of atmospheric environments and many corrosive media.

Medical grade 316L SS vm (vm □ vacuum melted, that means an additional decrease of the oxygen content in the steel), is presently used extensively in medicine for implants. This grade of stainless steel has been used to avoid and/or minimize the danger of pitting corrosion by making these high potentials at which the pitting occurs highly improbable, although this generally depends on the individual human being involved.

A variety of surface treatments are commonly performed on medical implant materials to promote corrosion resistance and biocompatibility [1,5,6,7,8,11,12,13,14,15]. For many years, electropolishing (EP) has been used to smooth the surface and to perform surface passivation on biomaterials. For a given material, the oxide properties are a function of the EP parameters such as applied current density, voltage, temperature, and the composition and concentration of the chemicals used. A stable oxide layer on the passivated metal surface obtained during EP will promote the corrosion resistance and biocompatibility of the implant material in physiological conditions. This passivity could be enhanced by modifying the thickness, morphology, and/or chemical composition of the surface oxide layer by different treatments [1,2,3,4,5,6,7,8,9,10,11,12,21,22,23,24,25,26,27]. 

Electropolishing is usually an advised treatment to finish metallic implants in view of taking advantage of the selective dissolution of elements constituting a particular metallic material. Selective dissolution enriches the passive layer in an element in which oxide is the most corrosion resistant and haemocompatible. The best example is 316L austenitic stainless steel. Electrochemical corrosion tests performed on standard electropolished and magnetoelectropolished 316L-stainless-steel samples in a 3% aqueous NaCl solution showed remarkable improvement in overall corrosion resistance [1,2,3,4,5,26,27]. These results include a decrease in nickel ion leaching. Further experiments are to be undertaken to determine the magnitude and mechanism of this phenomenon.

**Figure 1:**
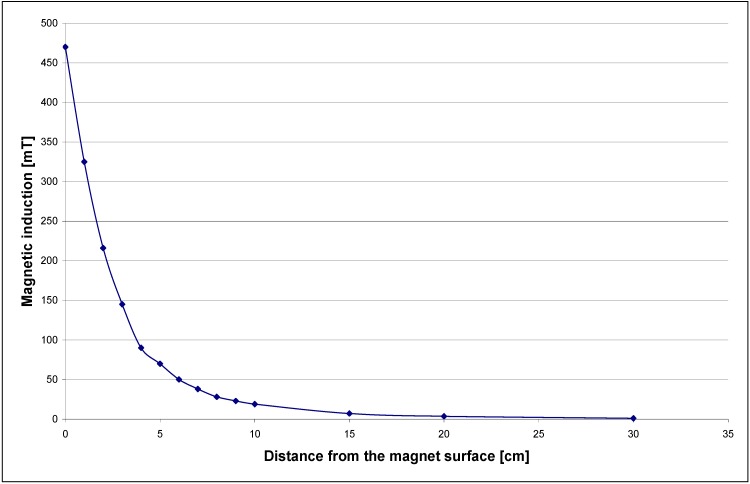
Characteristics of the neodymium magnet used for MEP system.

Electropolishing becomes even more complex if a magnetic field is introduced to the system [7,8,9,10,12,13,22,23,24,25,26,27,28,29]. Characteristics of the neodymium magnet used for the MEP system are presented in Figure 1. For the MEP both cylindrical (Figure 1) and ring magnets surrounding the electrolytic cell [1,2,3,4,5,26,27,28,29] have been applied. With the new electropolishing system, an externally applied magnetic field may enhance, or retard, the dissolution process. The electropolishing process is maintained under oxygen evolution to achieve an electropolished surface of the workpiece exhibiting reduced microroughness, better surface wetting and increased surface energy, reduced and more uniform corrosion resistance, minimization of external surface soiling and improved cleanability in shorter time periods. 

In this study the occurrence of the plateau and its level, and then the reference to the surface quality obtained has been presented. The results of the SEM, Auger spectroscopy, and XPS studies obtained herewith carried out on AISI 316L SS reveal very well the advantages of the magnetoelectropolishing process.

## 2. Experimental 

### 2.1. Set-up and parameters

First the polarization characteristics have been studied. The EP studies were carried out over a broad range of current density changes (up to 1,200 A/dm^2^), potential to over 10 V, up to 20 V vs. MSE (mercury-sulphate electrode] and electrolyte temperature (20 to 100 °C; with temperature control to ±1 °C). For the studies a proprietary sulphuric/orthophosphoric acids mixture electrolyte was used. 

**Figure 2:**
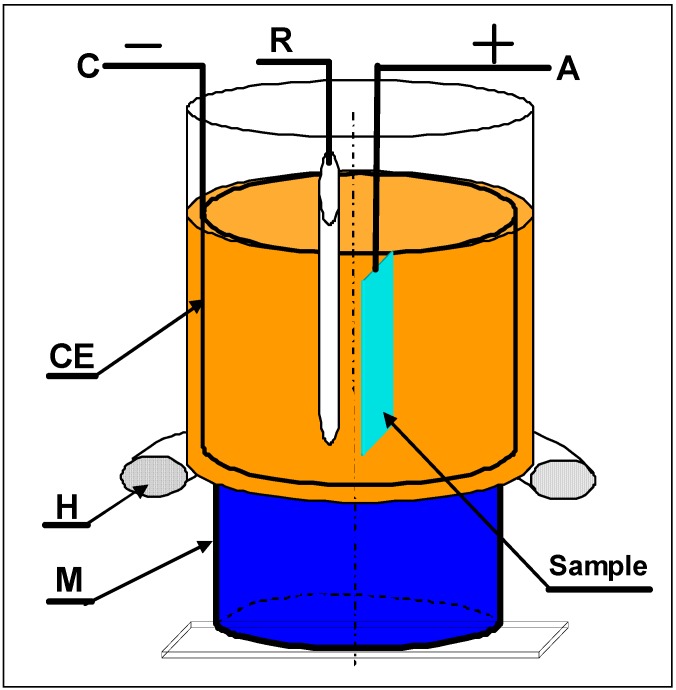
Magnetoelectropolishing MEP system used for investigation of polarization characteristics: A – anode, C – cathode, R – reference electrode, CE – counter electrode of cylindrical shape, CE – counter electrode, M – permanent magnet, H – heater.

For reference a mercury-sulphate Hg/Hg_2_SO_4_ electrode (MSE) was used in the electrolyte temperature range up to 60 °C. The electrode has been marked as REF601. The MSE reference element is based on Hg/Hg_2_SO_4_ and it uses a filling solution of saturated K_2_SO_4_. At 25 °C the REF601 potential versus a saturated calomel electrode is approximately 407 mV. This type of MSE reference electrode was successfully applied earlier by the authors [30] during investigation of electropolishing of carbon and tool steels using rotating disk electrodes. A stainless steel plate was used as a reference for the studies in temperature ranging above 60 °C, up to 120 °C. The set-up used for the MEP is presented in Figure 2.

The electrolytic polishing was performed both in the absence and in the presence of a magnetic field. A hallothrone device of type SMS-102 was used to measure the magnetic field strength. For the MEP experiments, a constant external magnetic field of 350 mT was applied to the EP system by a neodymium magnet. For both processes, conventional EP and MEP, the same type of a proprietary acidic electrolyte was used. The bath was unstirred during the process of MEP, and under a variety of electrochemical conditions during EP. 

During the investigation, measurement of polarization curves were performed on all stainless steel samples’ surfaces during both EP and MEP. The electrochemical system used for the investigation consisted of an ATLAS 98 potentiostat equipped with POL 98 software.

### 2.2. Material for samples

For these experiments, medical grade AISI 316L stainless steel was used (Table 1). Three sets of AISI 316L stainless steel samples of dimensions 30×40×1.2 mm were cut from the same sheet of metal. The first set consisted of three samples and they were polished with an abrasive grit paper up to No. 800 (MP – abrasive polishing). The second set of three samples were treated by a standard electropolishing (EP), the third set of three samples were electropolished with the same electrolyte composition in the presence of a magnetic field (MEP) [1,2,3]. 

**Table 1:** Chemical composition of AISI 316L SS used for the experiments [26].

Element	Content, wt%
Cr	17.07
Ni	10.26
Mo	1.97
Mn	1.68
Si	0.64
Cu	0.19
V	0.11
Co	0.04
C	0.03
Ti	0.03
P	0.024
Al	0.011
W	0.01
Sn	0.009
B	0.0048
S	0.004
N_2_	0.0431
Fe	BALANCE

The samples after mechanically abrasive polishing (MP) were used as a reference. For both electrochemical polishing processes, conventional/standard electropolishing (EP) and magnetoelectropolishing (MEP), the same type of a proprietary electrolyte was applied, being a mixture of sulphuric and orthophosphoric acids. Both electrolytic polishing processes, EP and MEP, were performed in a round, transparent electrochemical cell. The magnetoelectropolishing (MEP) was performed under identical conditions to those used for standard electropolishing process, except for the externally applied magnetic field. 

The magnets were magnetized by their thickness. The imposed magnetic field of about 350 mT was directed parallel to the electropolished workpiece surface (sample). During both electropolishing (EP) and magnetoelectropolishing (MEP) the bath was unstirred and temperature was kept within 60±1 °C. Prior to the MEP surface studies, the samples were thoroughly degreased in acetone. 

### 2.3. Corrosion behaviour studies

The corrosion resistance studies of the AISI 316L SS after three types of treatments – MP, EP, and MEP – were performed by investigating the Electrochemical Impedance Spectroscopy (EIS) and polarization curves in a typical Ringer’s solution at a room temperature (about 25 °C). The electrochemical system used for the corrosion measurements consisted of the ATLAS 98 potentiostat with the POL 98 software, current platinum electrode Ept-101 of surface area of 25 mm^2^, and a saturated calomel electrode (SCE) EK-101P used as a reference. The sample surface area exposed for the study each time was 2.01 cm^2^. Polarization curves were measured in the potential interval from −1,000 mV to 1,500 mV at a scan rate of 1 mV·s^-1^. The EIS results were obtained each time after holding the samples at open circuit potential (OCP) for 60 minutes. 

### 2.4. SEM, Auger, and XPS studies

SEM studies, Auger electron spectroscopy (AES) and XPS measurements were carried out on the MP, EP, and MEP 316L SS samples. The AES was performed under an electron energy of 3 keV. The XPS experiments were performed using a SCIENCE SES 2002 instrument. The studies were carried out under standard conditions within the binding energy BE range of −1,100 eV to 0 eV.

## 3. Results of the study

**Figure 3:**
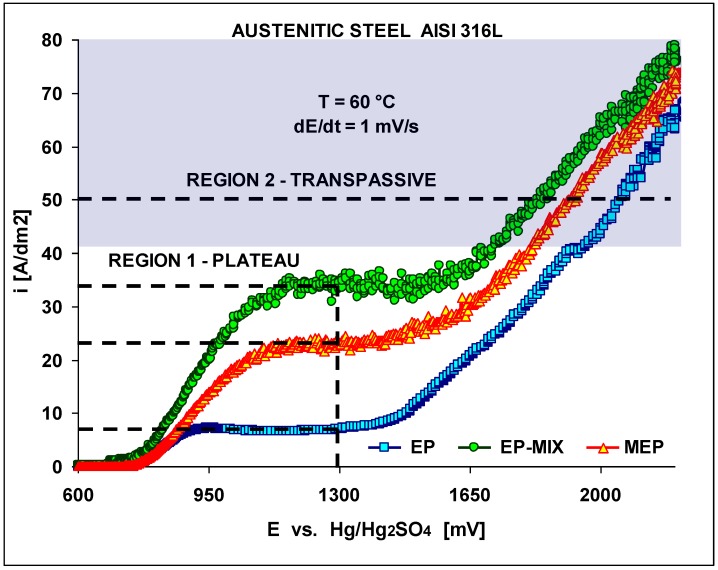
Electropolishing polarization characteristics obtained on AISI 316L SS dependent on treatment conditions (EP, EP with moderate mixing, and MEP) presenting two regions: 1 – plateau, 2 – transpassive. The semi-shadowed current density area of typical industrial applications has been indicated.

Initially the polarization characteristics under conventional (EP), and magnetoelectropolishing (MEP) conditions have been studied. The results are presented in Figure 3. The effects of the magnetic field were investigated in our studies. We began our studies with a conventional electropolishing EP to compare the results with the ones obtained under EP using a magnetic field. 

The polarization curves were obtained at an electrolyte temperature of 60±1 °C and a scan rate of 1 mV·s^-1^. The lowest cd plateau was obtained under a conventional EP in still electrolyte and it equaled about 8 A/dm^2^, then with a moderate mixing it rose up to about 33 A/dm^2^. Under MEP conditions (350 mT) the obtained plateau level was in between the two higher given values, and equaled about 23 A/dm^2^. In Figure 3 both, region 1, referring to the plateaux, and 2, transpassive region, have been presented, with the semi-shadowed area indicated and corresponding with the most often recommended for applications in industrial practice. 

**Figure 4:**
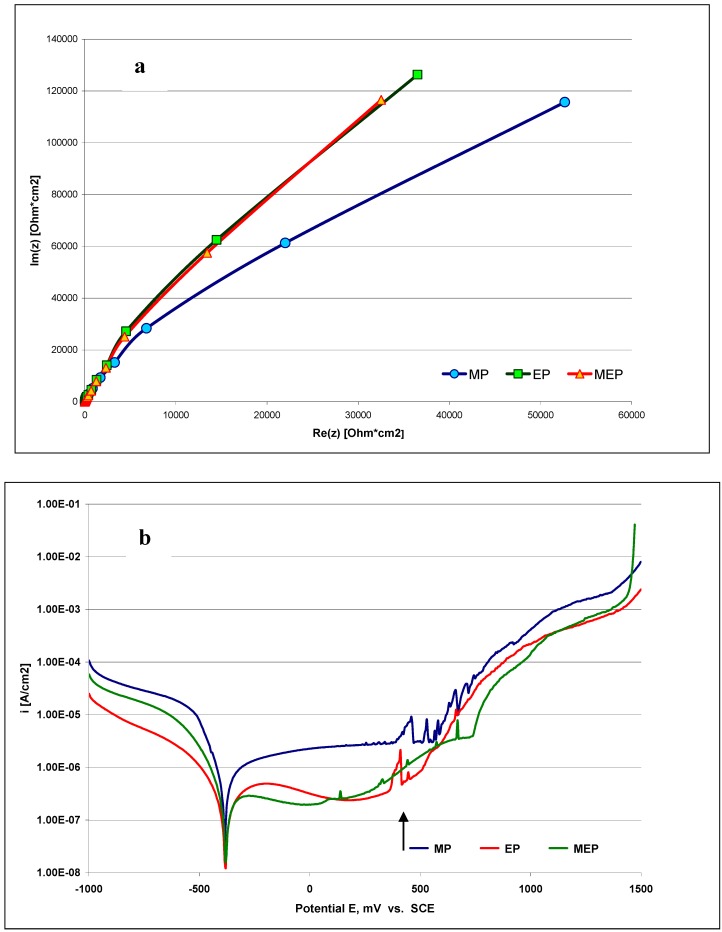
Electrochemical Impedance Spectroscopy results (Nyquist plots) of AISI 316L SS (a), and (b) polarization curves obtained on AISI 316L SS sample surface in Ringer’s solution; both after three types of treatments MP, EP, MEP. The arrow in Figure 4b indicates pitting development.

In Figure 4 the corrosion characteristics of the steel samples in a typical Ringer’s body fluid are presented. Figure 4a presents Nyquist plots and Figure 4b the polarization curves of AISI 316L SS after the three types of treatments – MP, EP, and MEP – indicating the most promising results were obtained after MEP. In Figure 4b the appearance of pitting has been indicated by an arrow, with the most pronounced being on the sample after MP, then after EP, and the least after MEP. 

**Figure 5:**
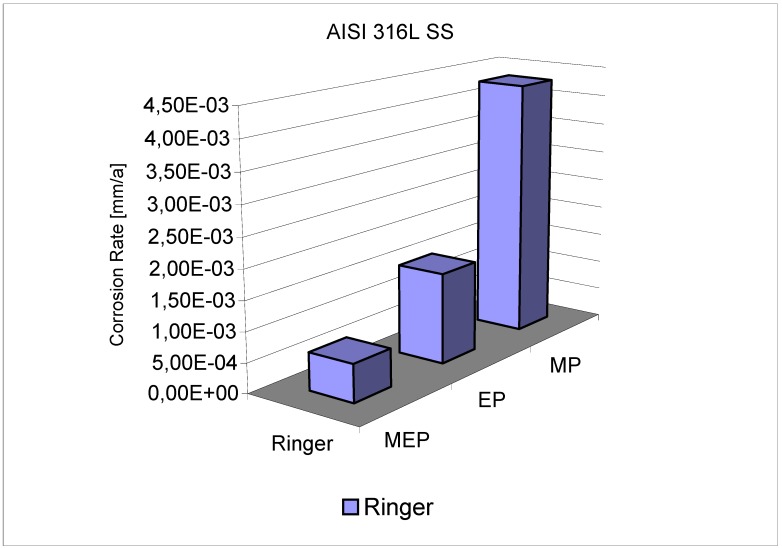
Comparison of corrosion rates of AISI 316L SS in Ringer’s solution after treatment by: MP – abrasive polishing (grit size 800), EP – standard electropolishing, MEP – magnetoelectropolishing [1,27].

In Figure 5 [1,27], the summary of the corrosion investigation results is presented. The corrosion rates in the typical Ringer’s solution, computed from the polarization curves after three treatments, MP, EP, and MEP, are presented. The results indicate the best corrosion resistance of 316L SS surface to be after MEP, and the biggest corrosion rate of the sample occurs after MP.

**Figure 6:**
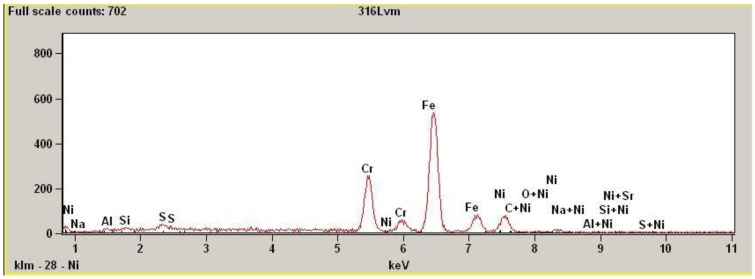
EDS results of 316L SS surface after magnetoelectropolishing (MEP), (save screen).

The scanning electron spectroscopy (SEM) investigation results are presented in Figure 6. In the picture the characteristic EDS data of 316L stainless steel composition profile is visible with prevailing peak of iron and chromium next to it. 

**Figure 7:**
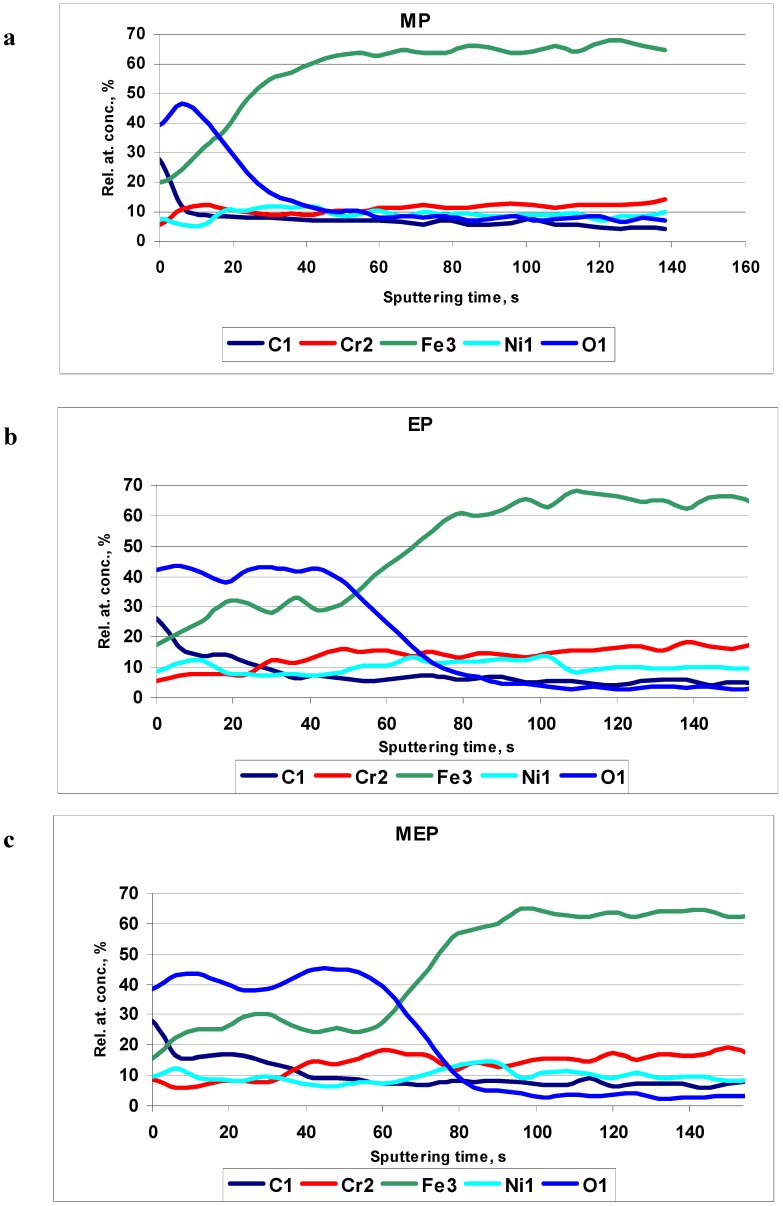
AES results of 316L SS surfaces after: (a) MP – abrasive polishing, (b) EP – standard electrolytic polishing, and (c) MEP – magnetoelectropolishing.

The Auger electron spectroscopy results are presented in Figure 7. In Figure 7a the typical Auger profiles after MP are presented. In the picture the relative concentration of iron is increasing very quickly with the sputtering time. This way the surface film thickness is rather small. Figure 7b indicates the oxide film after EP to be about three times higher than the oxide film after MP. After magnetoelectropolishing MEP (Figure 7c) the presented oxide/hydroxide film on the sample surface is even bigger than the film after a standard electropolishing EP. 

**Figure 8:**
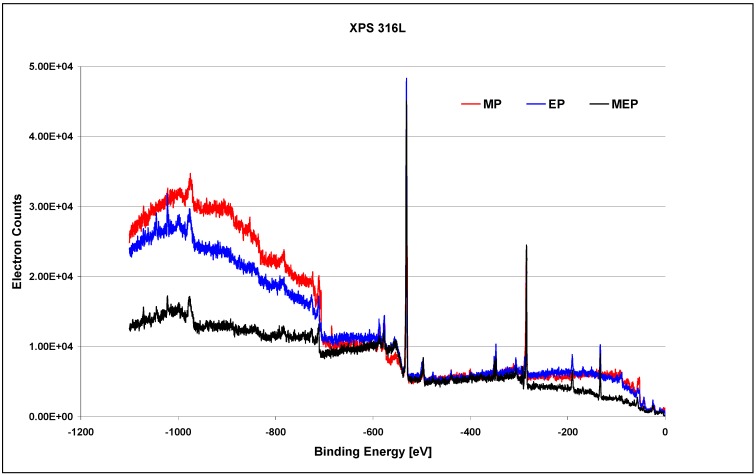
XPS results of 316L SS surfaces after: MP – abrasive polishing, EP – standard electrolytic polishing, and MEP – magnetoelectropolishing [26].

The XPS studies on 316L SS samples, after MP, EP, and MEP have been carried out within the binding energy of −1100 eV to 0 eV and the general results are presented in Figure 8 [26]. One may notice here different surface compositions after each of sample surface treatment, beginning from the sample after MP, then after EP, and next after MEP. 

The presented XPS results in the whole range of binding energy (Figure 8) after each of the finishing treatment: MP, EP, and MEP, may be analysed. After abrasive polishing (Figure 8 MP) the following elements dominate on the surface: oxygen, carbon, chromium and iron, and much lower peaks correspond to nickel, manganese, and molybdenum. After a standard electropolishing (Figure 8 EP) the level of oxygen increases (occurring mainly in the form of oxides), with such elements like calcium, sodium, and phosphorus being more noticeable, and with nickel vanishing in the background noise. The sample surface after magnetoelectropolishing (Figure 8 MEP) is also characteristic with high amount of oxides, with lower amounts of iron and chromium compounds, and with some peaks of non-magnetic elements, like phosphorus. 

**Figure 9:**
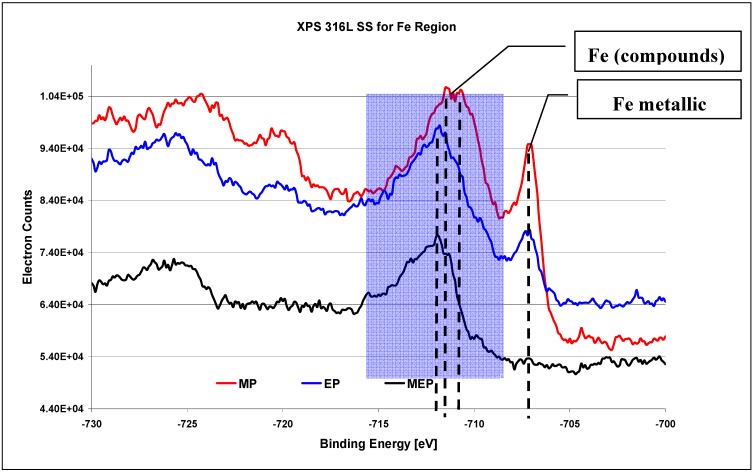
XPS results for Fe region of 316L SS surfaces after: MP – abrasive polishing, EP – standard electrolytic polishing, and MEP – magnetoelectropolishing [26].

The XPS results on 316L SS surface for Fe region (Figure 9) [26] indicate the occurrence of oxides Fe_x_O_y_ in each case, with varying amount of metallic iron after each consecutive kind of treatment: MP, EP, and MEP. The highest amount of free iron occurs after abrasive polishing (Figure 9 MP), lower after a standard electropolishing (Figure 9 EP), and no noticeable peak of iron is visible after magnetoelectropolishing (Figure 9 MEP). 

**Figure 10:**
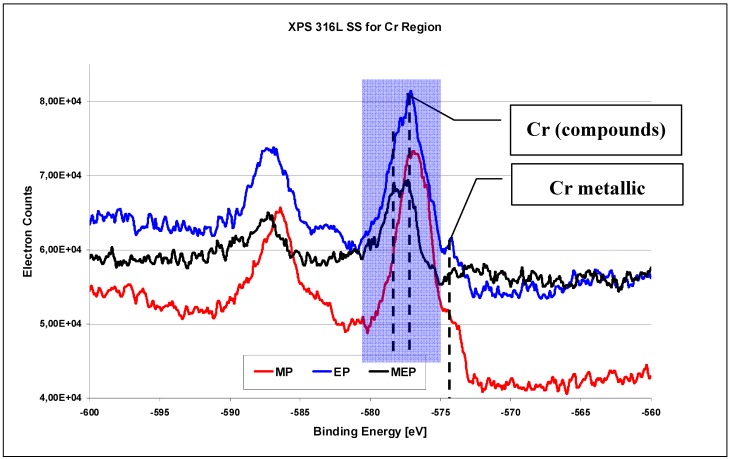
XPS results for Cr region of 316L SS surfaces after: MP – abrasive polishing, EP – standard electrolytic polishing, and MEP – magnetoelectropolishing [26].

The XPS investigation results on 316L SS surface for Cr region (Figure 10) [26] indicate the occurrence of oxides Cr_x_O_y_ in each case, with varying amounts of free chromium after each consecutive kind of treatment MP, EP, and MEP. The highest peak of free chromium occurs after a standard electropolishing (Figure 10 EP), some lower after abrasive polishing (Figure 10 MP), and no noticeable peak of free chromium is visible after magnetoelectropolishing (Figure 10 MEP). 

**Figure 11:**
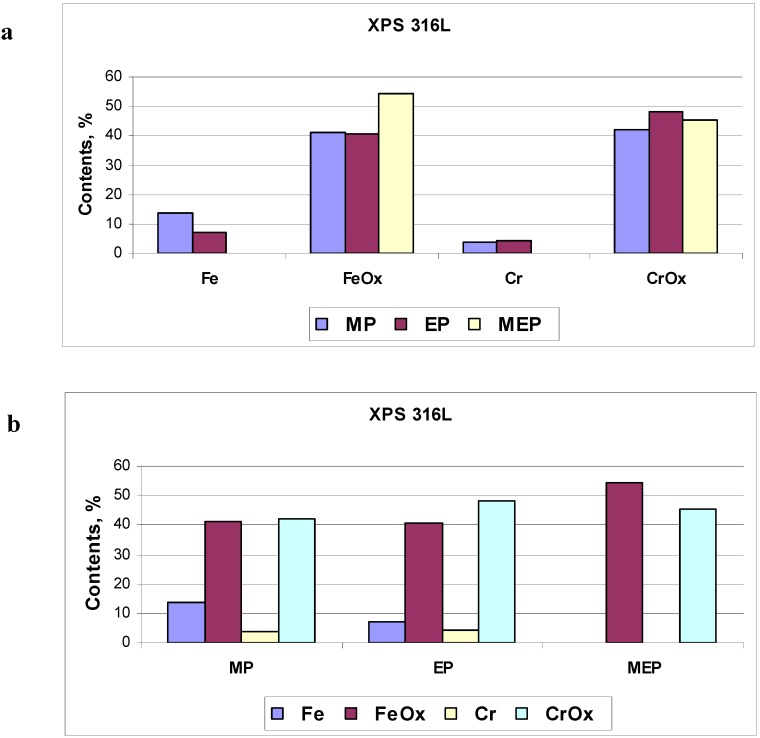
Calculated results of surface film composition on 316L SS sample: (a) revealing the presence or absence of free atoms and oxides, (b) dependent on kind of treatment, MP, EP and MEP.

The numerical amounts of oxides and pure metal atoms on 316L SS samples, after MP, EP, and MEP have been presented in Figure 11. In Figure 11a the percentage contents of particular elements of the surface film (Fe, FeO_x_, Cr, CrO_x_) is presented indicating no free metal atoms contents after magnetoelectropolishing MEP. In Figure 11b one may notice no free iron atoms on the sample surface after MEP, beside from no free chromium atoms independent on the kind of treatment. 

## 4. Discussion

Intially the polarization characteristics of the MEP process seem to be very similar to those in a standard/conventional electropolishing EP (Figure 2). The current density plateau level under MEP conditions (350 mT) occurred in between the two plateaux of a conventional EP in still electrolyte and with a moderate mixing. By electropolishing in the presence of a magnetic field (MEP) we have been able to improve many characteristics, specifically with regards to the increase of corrosion resistance in halide-containing environments like the Ringer’s body fluid (containing about 0.9% NaCl), (see Figure 4 and Figure 5). For those purposes, well-defined and recognized electrochemical procedures were used for the studies. It is probably connected with more uniform surface finishing and more advantageous surface roughness parameters, as presented in our earlier works [8,9,10] on one hand, and the specific morphology and oxide/hydroxide film composition on the other. This second set of properties has been the reason to undertake the present studies.

The AES results clearly show the difference between the composition of oxide film formed on MP, EP, and MEP samples (see Figure 7). One may notice the existent oxide/hydroxide film on the steel sample surface after MEP is bigger than the film after a standard electropolishing EP, and much bigger than after MP.

When analyzing the XPS iron region for binding energy BE equalling from −730 eV to −700 eV the progressive metallic iron washing out is observed starting from MP, through EP until MEP surfaces to a complete disappearance of that element after magnetoelectropolishing MEP (see Figure 9). On MP 316L SS surface the Fe_2_O_3_ oxides dominate for BE equaling −710.9 eV, and FeOOH compounds appear on EP and MEP 316L SS surfaces for BE equaling −711.8 eV. 

When analyzing the XPS chromium region for binding energies from −600 eV to −560 eV the metallic chromium peak is observed on MP and EP 316L SS surfaces for a binding energy of −574.4 eV (see Figure 10). On the other hand, no chromium peak is visible over the background noise on 316L SS surface after magnetoelectropolishing MEP. Here also the chromium oxides are of great interest. After MP and EP of 316L SS samples generally Cr_2_O_3_ oxides are apparent for binding energy equaling −576.8 eV. After MEP of 316L SS sample the hydroxides Cr(OH)_3_ occur for binding energy equaling −577.3 eV, and some non-stoichiometric CrO_x_ oxides occur, with x below 3, for binding energy equaling −578.3 eV. The occurrence of oxides and hydroxides of chromium and iron on 316L SS surface layer after electrolytic polishing (EP) were reported also by Selvaduray and Trigwell [14], and Kerber and Tverberg [31]. Our earlier studies carried out on 316L SS surface after EP and MEP by Auger electron spectroscopy indicated on decreasing amount of Fe against Cr. After a typical abrasive polishing (MP) a Cr/Fe ratio indicates 0.7-0.74, whereas after EP it is usually over 1, to about 1.2. The XPS results obtained on AISI 316L after MEP indicated the Cr/Fe ratio to be over 1.7 [1,22,27], and our recent investigations show the ratio to reach even 3. It is much better than after a standard EP and it is believed to be the reason for improved corrosion behaviour. On the other hand, the reported Cr/Fe ratio result after EP and additional operation relying on passivation in nitric acid showed at maximum of 2.3 [31].

The magnetoelectropolishing process carried out with oxygen evolution contradicts, to some degree, all three of the established electropolishing theories. When the electropolishing process is carried out under the oxygen evolution regime, the properties of oxygen and its behaviour in a magnetic field are the critical factors. Oxygen is a paramagnetic element with two unpaired electrons that are attracted and aligned by a magnetic field. It is believed that some oxygen molecules, which are released during decomposition of the oxide layer, escape to the electrolyte. Others are attracted by the nonzero magnetic field. Those attracted oxygen molecules likely migrate toward the metal surface through the cyclically oxidized surface, or through vacancy sites, and dissociatively adsorb. This makes the oxide-hydroxide layer more compact and homogenous and thus more difficult to dissolve. The dissociatively adsorbed oxygen must be responsible for the decrease in current density and, consequently, for the rate of dissolution of electropolished material.

The characteristic feature of MEP process of steel surface is eliminating free metals from the film (see Figure 11) which has not been possible in any other surface treatment (MP, and/or EP). Instead, only respective oxides have been revealed on samples after magnetoelectropolishing. An additional enrichment of the surface layer in oxides may be obtained after post-electropolishing passivation process. It would be interesting to compare this magnetically electropolished (MEP) surface with a nitric or citric passivated surface, and such a research is planned as the next step of the study.

These Auger and XPS studies have evidenced that changes in composition of the surface film appear to have a decisive influence on the 316L SS corrosion behaviour. Thus, presented in other authors’ papers [1,26,27,28], improved the corrosion behaviour well coincides with the contribution of chromium and iron oxides content in the surface film after MEP treatment.

## 5. Conclusions

The investigation results presented show the differences both in polarization characteristics obtained during EP and MEP, corrosion resistance, and in the surface film morphology and composition of a medical grade 316L stainless steel between mechanically polished MP, standard electropolished EP, and magnetoelectropolished MEP surfaces. Thus modified 316L stainless steel surface film composition well coincides with the results showing improved corrosion behaviour of the samples obtained after three treatments: MP, EP, and MEP. The following additional conclusions may be drawn from the studies:

(1) the plateau in the course of polarization curve during MEP with 350 mT was found to be between the plateaux of standard EP carried out in still and moderately mixed electrolyte.
(2) the treated 316L SS samples indicate improving corrosion resistance in halide media from the least, after MP, through EP, to the best after MEP.
(3) electrolytic polishing of 316L SS in a magnetic field (MEP) results in disappearance of both metallic Cr and Fe and formation of oxides and hydroxides of these metals.
(4) a noticeable change in chemical composition on the studied 316L SS surfaces from Fe_2_O_3_ to FeOOH and from Cr_2_O_3_ into Cr(OH)_3_ and CrO_x_ (with x being below 3, not reaching CrO_3_) is apparent on the samples after MEP.
(5) the magnetic field applied for electropolishing of 316L SS results in selective leaching of magnetic elements like Fe and Ni from the non-magnetic austenitic steel.

The modified 316L SS sample surface after MEP process has indicated a decreased corrosion rate in Ringer’s body fluid. All used surface study techniques, SEM, the Auger spectroscopy, and XPS examinations revealed advantageous changes in the surface film formed on AISI 316L SS resulting from incorporation of the magnetic field into electropolishing process. Magnetoelectropolishing seems to be a reasonable process for using it to modify metal surface film composition and improve performance of 316L SS biomaterial.
